# State variation in neighborhood COVID-19 burden across the United States

**DOI:** 10.1038/s43856-024-00459-1

**Published:** 2024-03-01

**Authors:** Grace A. Noppert, Philippa Clarke, Andrew Hoover, John Kubale, Robert Melendez, Kate Duchowny, Sonia T. Hegde

**Affiliations:** 1 Institute for Social Research, University of Michigan, Ann Arbor, USA; 2 Department of Epidemiology, Johns Hopkins University, Baltimore, USA

**Keywords:** Viral infection, Epidemiology

## Abstract

**Background:**

A lack of fine, spatially-resolute case data for the U.S. has prevented the examination of how COVID-19 infection burden has been distributed across neighborhoods, a key determinant of both risk and resilience. Without more spatially resolute data, efforts to identify and mitigate the long-term fallout from COVID-19 in vulnerable communities will remain difficult to quantify and intervene on.

**Methods:**

We leveraged spatially-referenced data from 21 states collated through the COVID Neighborhood Project to examine the distribution of COVID-19 cases across neighborhoods and states in the U.S. We also linked the COVID-19 case data with data on the neighborhood social environment from the National Neighborhood Data Archive. We then estimated correlations between neighborhood COVID-19 burden and features of the neighborhood social environment.

**Results:**

We find that the distribution of COVID-19 at the neighborhood-level varies within and between states. The median case count per neighborhood (coefficient of variation (CV)) in Wisconsin is 3078.52 (0.17) per 10,000 population, indicating a more homogenous distribution of COVID-19 burden, whereas in Vermont the median case count per neighborhood (CV) is 810.98 (0.84) per 10,000 population. We also find that correlations between features of the neighborhood social environment and burden vary in magnitude and direction by state.

**Conclusions:**

Our findings underscore the importance that local contexts may play when addressing the long-term social and economic fallout communities will face from COVID-19.

## Plain language summary

A lack of data on the geographic location of COVID-19 cases in the U.S has limited our ability to examine how COVID-19 burden has been distributed across neighborhoods within U.S. states. It may be that certain neighborhoods have borne a disproportionate burden of COVID-19 and are more likely to experience greater long-term social and economic consequences from the pandemic. We used data from 21 states to examine the distribution of COVID-19 cases across neighborhoods and states in the U.S. We find that the distribution of COVID-19 varies widely both within neighborhoods in a state, and between states. We also find that the features of the neighborhood social environment that are correlated with neighborhood COVID-19 burden vary by state. Our findings show that the local neighborhood may play an important role in addressing long-term social and economic consequences from COVID-19.

## Abstract (web summary)

Noppert et al. examine how the distribution of COVID-19 cases at the neighborhood-level varies significantly within and between states in the United States. The authors report that correlations between features of the neighborhood social environment and COVID-19 burden vary in magnitude and direction by state.

## Introduction

In the United States (U.S.), the COVID-19 pandemic has affected nearly every American and nearly every part of American life. However, the burden of COVID-19 has not been equally distributed across the U.S and globally. A recent study by Bollyky et al. provides compelling evidence for the stark differences in the distribution of COVID-19 cases and deaths across states within the U.S., as well as the link between those state-level differences and socioeconomic and demographic state characteristics. The authors reported up to a four-fold difference in age- and comorbidity-standardized COVID-19 death rates across states in the U.S.^1^ Moreover, infections and deaths were disproportionately clustered in states with lower levels of education, higher levels of poverty, limited access to healthcare, and lower levels of self-reported trust in one another^1^. This report follows a 2021 study that found that individuals living in U.S. states with high levels of pre-pandemic poverty and a greater proportion of non-Hispanic Black individuals experienced a greater number of COVID-19 hardships including food insufficiency, loss of income, unemployment, and housing instability, and that racialized minorities had a slower recovery from these hardships than their white counterparts^2^.

While variation in disease risk and post-pandemic socioeconomic repercussions between states has been well documented, a growing number of studies have demonstrated such heterogeneities also exist within states, and specifically between neighborhoods within states^3–15^. Understanding local trends is essential for not only quantifying but intervening on the growing social and economic inequities that are a consequence of the uneven burden of COVID-19. Neighborhoods are a source of both risk and resilience to COVID-19 and its long-term sequelae due to their social and economic characteristics (e.g., crowding, housing density, affluence, business types, and political partisanship)^16–22^. Since the start of the COVID-19 pandemic, socioeconomically disadvantaged neighborhoods have faced substantially greater population losses, economic hardships, and business closures compared to less disadvantaged neighborhoods, particularly those neighborhoods with a greater share of working-aged adults. For example, in the early months of the pandemic, a multi-state study of COVID-19 prevalence by ZIP code found a higher burden of disease in socioeconomically disadvantaged ZIP codes in Illinois and Maryland^3^.

Despite initial evidence indicating neighborhood and state contexts play an important role in shaping COVID-19 risk and resilience, two major gaps in both data availability and research have hampered efforts to fully understand neighborhood variation within states in the U.S. First, there has been lack of fine-scale, spatially-resolute COVID-19 case data made available publicly for analysis across the U.S. The national efforts to collate spatially-referenced data have largely focused on the county- or state-level (e.g., Centers for Disease Control and Prevention COVID-19 Data Tracker or the Johns Hopkins University Coronavirus Resource Center, or the Institute for Health Metrics and Evaluation’s (IHME) COVID-19 modelling database)^1,23–27^. There have been several initiatives that seek to understand neighborhood trends but have focused on select cities only. For example, The Big Cities Health Coalition (BCHC) created a dashboard where the distribution of COVID-19 cases across neighborhoods (i.e., ZIP code tabulation areas) could be examined and linked with relevant social indicators for 35 U.S. cities^28^. In another example, the City Health Dashboard initiative provided small-scale, spatially-referenced data on a range of health, social, and demographic metrics for over 750 small and large cities in the U.S.^29^, which at one point included a neighborhood-level COVID-19 risk index^30^.

Second, while some studies have examined neighborhood variation in COVID-19 burden, they are limited to cities, thereby omitting data from geographic areas beyond the chosen urban settings. For example, studies that have examined finer spatial levels (e.g., ZIP code and/or census tract) have been limited to a single city, region, or state^3–5,8–14,31–47^. Some of these studies have examined a group of cities, regions, or states, including the BCHC initiative noted above but none have employed an expansive effort to look at trends across diverse regions of the U.S.^3,10,31,36,38,45^ As a result of both the data and research gaps described above, the analyses that have been conducted examining neighborhood-level variation and state differences in neighborhood-level variation have been biased towards cities lending little insight into what is happening among rural populations. Several studies conducted early in the pandemic documented temporal and spatial differences in COVID-19 burden between urban and rural areas^48^ with rural areas continuing to experience spikes in COVID-19 cases when cases were receding in urban areas^49^. Further, these trends extend beyond the U.S. setting; studies in both the U.S. and U.K. have documented the unequal toll the pandemic and its aftermath continue to have on rural communities compared to urban communities^50–52^. Taken together, these studies highlight the need for rural communities to be included in both our data and models to understand the predictors of COVID-19 burden and its consequences.

To directly address these gaps, we launched the COVID Neighborhood Project (CONEP) in 2021. CONEP is a repository of locally-referenced (census tract or ZIP code tabulation area (ZCTA)) COVID-19 case data from April 2020 to April 2022 for the U.S. The repository currently includes cumulative case data for 21 states in all five regions of the U.S. (West, Southwest, Midwest, Northeast, and Southeast). Data collation for the remaining states is ongoing. The novelty of this resource has enabled a closer examination of both state- and neighborhood-level variation in COVID-19 burden across a more expansive portion of the U.S., including both urban and rural areas. These local patterns, including in both rural and urban areas across the U.S., are critical to our understanding of which communities will continue to face short-term and long-term health, social and economic consequences from COVID-19, while laying the groundwork for future pandemic preparation.

In this paper, we leverage data from CONEP to illustrate how neighborhood-level COVID-19 infection burden varies between states and examine the neighborhood social characteristics that are correlated with infection burden. We address a critical gap in the literature with the following research questions: (1) How is COVID-19 distributed across neighborhoods (i.e., ZCTAs or census tracts) within states, and is the neighborhood distribution of COVID-19 burden similar between states? (2) Within each state, what features of the neighborhood social environment are correlated with neighborhood COVID-19 burden? We find that the distribution of COVID-19 at the neighborhood-level varies both within and between states. Some states have a more homogenous distribution of COVID-19 compared to other states in which there is a wide variation in COVID-19 burden within the state. We also find that the neighborhood social factors that are correlated with neighborhood COVID-19 burden vary both in magnitude and direction by state.

## Methods

### The COVID Neighborhood Project

We launched CONEP in 2021 to address the need for fine-scale spatially resolute COVID-19 case data for the entire U.S. We contacted health departments in all 50 U.S. states. Initial contact consisted of calls, emails, and data portal requests asking for fine-scale COVID-19 case data. For some states, census tract or ZIP code data were publicly available. Others required a Freedom of Information Act (FOIA) request. Following request edits, resubmissions, and the eventual approval, we were sent data through a secure email.

### COVID-19 case data and neighborhood data

We used COVID-19 cases at the ZIP-code level for sixteen states, and census tract-level for five states. Data were collected from state health departments throughout the summer and fall for both 2021 and 2022. Data were collected in a cumulative format. State COVID-19 cases were defined as they were by each respective state health department, most commonly as the sum of both laboratory confirmed and probable cases (1–3). Most states did not disaggregate their case data by time period.

We collected both census tract and ZIP code level data. For those states with ZIP code level data, we used a cross-walk to merge ZIP codes into ZIP Code Tabulation Areas (ZCTA)s. ZIP codes are designated by the U.S. Postal Service and used to identify postal delivery routes. Therefore, they do not represent a spatial area. ZCTAs are generated by the U.S. Census Bureau and are generalized representations of ZIP codes. Methods used to create ZCTAs are detailed elsewhere^53^. Of note, ZIP-code level data were not available in Florida past 23 June 2021.

The study was approved by the Health and Behavioral Sciences Institutional Review Board at the University of Michigan (IRB Approval Number: HUM00202190). Data use agreements were done on a state-by-state basis (see Data and Materials availability statement below). As this study involves only the secondary analysis of de-identified data, informed consent was not required.

Estimates of the neighborhood social and physical context from the National Neighborhood Data Archive (NANDA)

The primary aim of the current investigation was to report state-level patterns in the distribution of COVID-19 burden at the neighborhood level. However, the ultimate goal of CONEP is to examine how features of the social and physical environment have shaped COVID-19 burden throughout the pandemic, providing a roadmap for addressing the long-term consequences certain communities will face. As previous studies of both COVID-19, as well as many other infectious diseases, have consistently demonstrated the importance of neighborhood SES in determining the distribution of infectious disease burden^3,24,54–56^, we focused on two specific measures of neighborhood SES: neighborhood disadvantage and neighborhood affluence. We also employed measures of neighborhood-level rurality, population density, and county-level political partisanship.

We obtained measures of the neighborhood context from NANDA, a publicly available repository of curated measures of social and physical environment context across the US.^57^.

Neighborhood disadvantage is an analytically derived index and is the mean of four variables collected as part of the American Community Survey (ACS): proportion of female-headed households with children, proportion of households receiving public assistance income or food stamps, proportion of families with income below the federal poverty level, and proportion of the population aged 16 years and older that are unemployed^57^. Mean scores range from 0-100. Disadvantaged neighborhoods tend to have fewer resources (e.g., healthy food stores, well-maintained parks, good schools, quality medical care)^20,58^ and are often vulnerable to disinvestment and environmental hazards^59^. These measurements were based on 2017 ACS data with the exception of New Mexico which was based on the 2020 ACS.

Neighborhood affluence is the mean of three variables from the ACS: percent of household with income greater than $75K per year, percent of the population over the age of 16 employed in professional or managerial occupations, and percent of the population with a Bachelor’s Degree or higher^57^. Mean scores range from 0-100. Higher values indicate a more affluent neighborhood. Affluent neighborhoods are likely to attract a set of institutions (e.g., food stores, places to exercise, well-maintained buildings, and parks) that foster a set of norms (e.g., an emphasis on exercise and healthy diets) conducive to good health^60^. Both from a theoretical and analytical standpoint, neighborhood affluence is distinct from neighborhood disadvantage^57^. Neighborhood affluence is associated with higher levels of social control and leverage over local institutions that can foster social environments that facilitate health^16^. These measurements were based on 2017 ACS data with the exception of New Mexico which was based on the 2020 ACS.

Neighborhood rurality was defined based on the 2010 rural-urban commuting area (RUCA) codes^61^. The RUCA was defined at both the census tract and ZCTA level. In the correlation analyses, we used an ordinal RUCA code in which higher values indicate more rural areas. In the univariate analyses, we used a four-level variable classifying neighborhoods according to their RUCA code as metropolitan, micropolitan, small town, or rural. Of note, the 2010 RUCA was not available for New Mexico as they used 2020 geography to define their census tracts and the 2020 RUCA codes are not available yet.

Neighborhood population density is an indicator of the number of persons per square mile in the census tract and/or ZCTA^57^. We included neighborhood population density to proxy the potential for exposure to the SARS-COV-2 pathogen within a given neighborhood with the hypothesis that those neighborhoods with higher population density would have higher COVID-19 case counts. Given that contact with an infectious pathogen is a necessary cause of infectious disease, we use neighborhood population density as an imperfect proxy of the probability of encountering an infectious case of COVID-19^55^.

We defined county-level political partisanship with a continuous measure that indicates the mean percent of votes cast for Republican candidates in presidential and senate races from 2012 to 2018^62^. In univariate analyses, we categorized the variable such that a one-unit increase in the political partisanship variable was equivalent to 10% increase in Republican votes. We linked ZCTAs and census tracts to the respective counties in which they resided. For ZCTAs that spanned two counties, we chose the county that had the greatest proportion of the ZCTA. Further, we use a measure of county-level political partisanship to begin to understand the complex role that political ideology has had in shaping COVID-19 testing, vaccination access, and mitigation strategies in the US^63,64^.

### Statistical analyses

We first calculated the cumulative COVID-19 case count per 10,000 population for each state. We then calculated the median case count per 10,000 and coefficient of variation (CV) for ZCTAs or census tracts within states cumulatively for the entire time period for which the state reported data. The CV is calculated by taking the standard deviation and dividing it by the mean in each state.

We estimated the correlation between each neighborhood variable and COVID-19 burden for each state. We then constructed a series of univariate Poisson regression models to estimate the association between each neighborhood factor and the incidence rate ratio (IRR) of COVID-19 cases for neighborhoods within each state. The total population of the neighborhood served as the offset term in the models. We used generalized estimating equations with robust standard errors to account for clustering of census tracts and ZCTAs in counties.

All statistical analyses were carried out in Stata/mp 17.0 and 18.0.

## Results

### Cumulative case counts are heterogenous between states

Of the spatially-referenced COVID-19 case data from 21 states, 5 states have data at the census-tract level and 16 states have data at the ZIP code level (Table 1). We used a cross-walk to convert the ZIP codes to Zip Code Tabulation Areas (ZCTAs) which is further described in the methods. Case data generally span 2021-2022, although there is state variability in the specific temporal reporting coverage (Table S1). We calculated the cumulative case count (census-tract or ZCTA) per 10,000 population for the entire time period (Table 1). Florida has the lowest cumulative case count of 1108 cases per 10,000 population and Wisconsin the highest with 3142 cases per 10,000 population for the two-year period (Table 1). Multiple states have spatial units with sub-populations that are missing case data (Table S1). See Supplementary Figs. S1–3 for depictions of the distribution of COVID-19 across neighborhoods within states.

**Table 1:** States for which data was obtained, the corresponding spatial resolution, and cumulative COVID-19 case counts per 10,000 population (April 2020-April 2022)

State	Spatial resolution	Cumulative case count	Cumulative case count per 10,000 population	Median (CV) by spatial resolution per 10,000 population
Northeast				
Delaware	Census tract	279,643	2589	3071.00 (0.19)
Maine	ZCTA	263,135	1978	1692.88 (0.53)
Maryland	ZCTA	1,137,968	1898	1785.71 (0.32)
New York	ZCTA	4,971,935	2511	2325.12 (0.39)
Pennsylvania	ZCTA	2,351,193	1838	1718.27 (0.5)
Rhode Island	Census tract	289,854	2744	2701.03 (0.24)
Vermont	ZCTA	101,968	1632	810.98 (0.84)
Southwest				
Arizona	ZCTA	1,972,243	2896	2520.20 (0.5)
New Mexico	Census tract	499,280	2381	2237.81 (0.44)
Oklahoma	ZCTA	1,035,202	2657	2491.10 (0.33)
West				
Nevada	ZCTA	686,165	2376	2008.01 (0.49)
Oregon	ZCTA	738,732	1835	1826.76 (0.3)
Southeast				
Florida	ZCTA	2,246,928	1108	966.55 (0.45)
Louisiana	Census tract	1,037,714	2225	2200.34 (0.21)
North Carolina	ZCTA	2,701,403	2687	2444.22 (0.21)
Virginia	ZCTA	1,743,838	2084	2052.05 (0.43)
Midwest				
Illinois	ZCTA	3,454,346	2687	2774.27 (0.26)
Indiana	ZCTA	1,542,971	2333	2354.78 (0.21)
Minnesota	ZCTA	1,584,267	2885	2695.07 (0.25)
Ohio	ZCTA	2,722,140	2345	2268.35 (0.25)
Wisconsin	Census tract	1,815,699	3142	3078.58 (0.17)

*CV* coefficient of variation.

### Neighborhood-level trends in COVID-19 burden differ by state

We calculated the median case count per 10,000 population and the coefficient of variation (CV) by ZCTA and census tract (Table 1). The median case count per 10,000 population at the neighborhood-level mirrors the trends of the cumulative case count per 10,000. The CV, however, gives an indication of the neighborhood-level variability in mean case counts within each state, and there are notable state-level differences in the CV of the neighborhood-level cases counts. For example, the highest CV’s are observed in Vermont (CV = 0.84) and Maine (0.53), which demonstrates a higher within-state variability in the mean of COVID-19 cases at the neighborhood-level. In contrast, the low CVs for states such as Wisconsin (CV = 0.17) and Delaware (CV = 0.19) suggest a more homogenous spread of COVID-19 cases across neighborhoods within those respective states.

### Correlations between neighborhood social factors and neighborhood COVID-19 burden differ within and between states

**Fig. 1:**
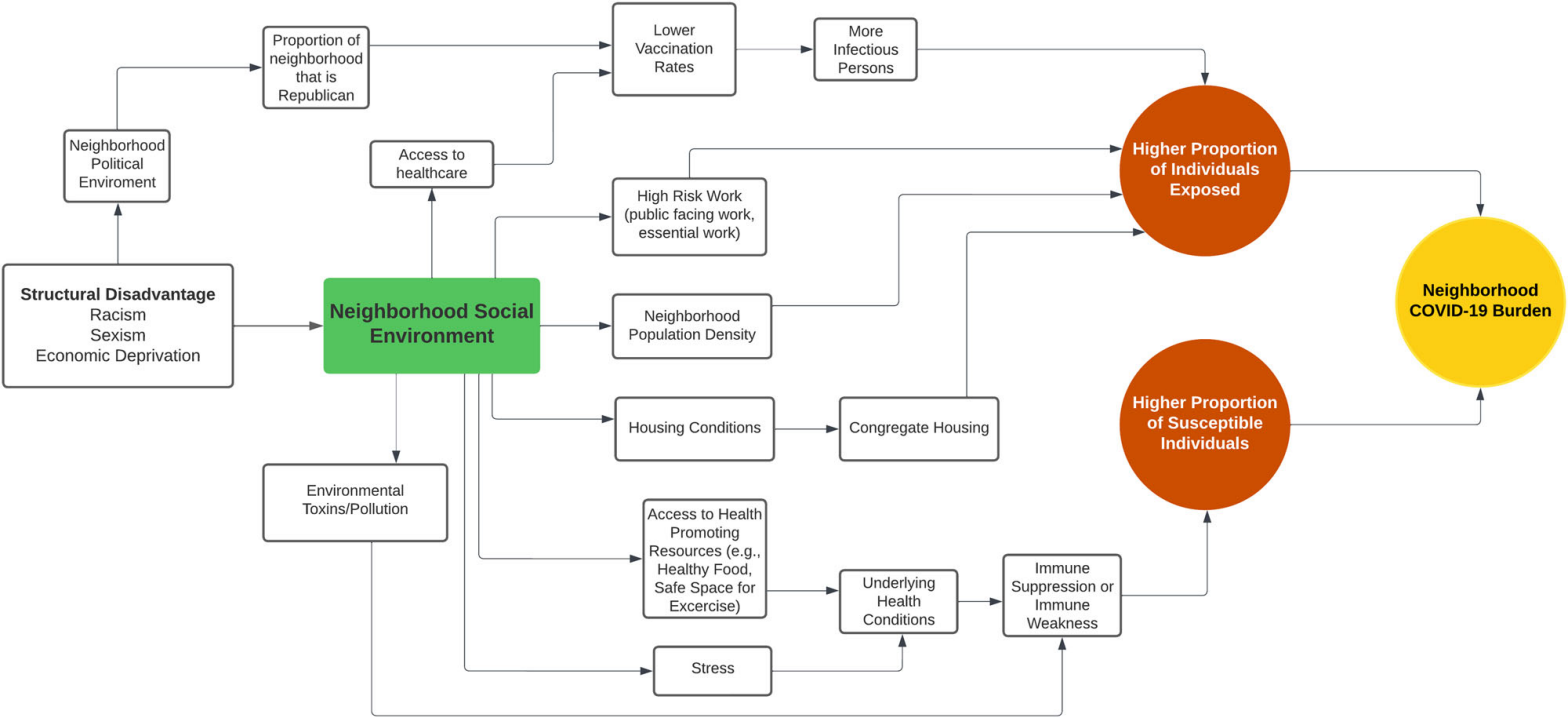
Conceptual Diagram. Conceptual diagram describing features of the neighborhood social environment may influence the neighborhood burden of COVID-19.

To address the second research question, whether there are features of the neighborhood social environment that are correlated with COVID-19 burden within a state, we used a series of univariate Poisson regression models to estimate the incidence rate ratio (IRR) of case counts cumulatively per 10,000 population for each neighborhood within each state. We used the recent framework by Noppert, Hegde, and Kubale (2022) to select features of the neighborhood environment that may be particularly relevant for understanding COVID-19 burden, and that may also lend themselves to intervention^65^. Their framework posits that infectious disease burden is a function of two primary pathways which may operate at both the individual- and neighborhoods-level: exposure, factors that increase the probability of exposure to an infectious pathogen, and susceptibility, factors that increase the likelihood of being infected if exposed. See Fig. 1 for the conceptual diagram used to guide the selection of neighborhood social and physical features.

The framework describes the neighborhood environment as one mechanism through which structural disadvantage operates to affect risk of infectious disease^65^. The neighborhood environment, as described in this manuscript, is a broad indicator that includes aspects of both the physical and social environment. For the current investigation, we chose to operationalize the neighborhood environment by first selecting two widely used measures of the neighborhood social environment: neighborhood disadvantage and neighborhood affluence. We modeled neighborhood disadvantage and neighborhood affluence as two separate factors given that they capture two distinct concepts of neighborhood socioeconomic status (SES), which is described further in the Methods section below. Briefly, neighborhood SES is essential to understanding the burden of infectious diseases. Low SES neighborhoods tend to have fewer health resources (i.e., hospitals, grocery stores, pharmacies), a higher proportion of their populations employed in lower wage jobs, or jobs classified as essential work with less ability to work from home or take sick leave when needed, and a higher proportion of the population utilizing public transportation^14,19,66^. All of these factors increase the probability that an individual is more likely to come into contact with an infectious pathogen, with inadequate protections to prevent infection^67^. Moreover, there is an inherent stress to living in a low SES neighborhood that may itself make an individual more susceptible to an infectious disease^68,69^. To begin to capture these dynamics, we examined both neighborhood disadvantage, a reflection of material access to resources, and neighborhood affluence, reflection of the power a community has to advocate for resources.

We also examined correlations between case counts and rurality (census tract or ZCTA-level) and political partisanship at the county-level. In the current U.S. context, political partisanship has been shown to be an indicator for the willingness to engage in public health prevention behaviors including vaccination^64,70^. We examined correlations with a rurality index (rural-urban commuting area code (RUCA)) given that multiple recent studies have demonstrated how rural and urban communities have fared differently throughout the pandemic^49,52^. Finally, we examined correlations with neighborhood population density as it is conceptualized as a key mechanism that can increase the probability of exposure to an infectious pathogen.

We observe univariate associations between neighborhood affluence and neighborhood COVID-19 burden for many states (Tables 2–6), however, the magnitude and direction of the association differ widely between states. In 52% of states, neighborhood affluence is statistically significantly, negatively correlated with neighborhood COVID-19 burden (Table S2). In these states, neighborhoods in the highest quartile of affluence (Q4; i.e., the most affluent) have significantly lower COVID-19 burden compared to neighborhoods in the lowest quartile of affluence (Q1). For example, in Rhode Island neighborhoods, we observe a 30% lower (*b* = 0.70, 95% CI: 0.65–0.76) incidence rate of COVID-19 comparing neighborhoods in the highest quartile of affluence (Q4) to neighborhoods in the lowest quartile of affluence (Q1). In New Mexico, neighborhoods in the highest quartile of affluence (Q4) have a 29% lower (*b* = 0.71, 95% CI: 0.62–0.82) rate of COVID-19 compared to neighborhoods in the lowest quartile (Q1).

**Table 2:** Results of the univariate regression results estimating the association between each neighborhood factor and neighborhood COVID-19 burden for the Northeastern United States

Northeast							
	Delaware	Maine	Maryland	New York	Pennsylvania	Rhode Island	Vermont
	IRR [95% CI]	IRR [95% CI]	IRR [95% CI]	IRR [95% CI]	IRR [95% CI]	IRR [95% CI]	IRR [95% CI]
Neighborhood characteristic							
Neighborhood affluence							
Q1 (ref.)	Ref.	Ref.	Ref.	Ref.	Ref.	Ref.	Ref.
Q2	0.98 [0.91–1.06]	0.96 [0.86–1.06]	0.96 [0.89–1.03]	1 [0.97–1.04]	0.94 [0.85–1.04]	0.86*** [0.8–0.93]	1.67 [0.83–3.38]
Q3	0.95 [0.89–1.02]	0.91* [0.83–1]	0.92+ [0.84–1.01]	1.04 [0.97–1.11]	0.99 [0.91–1.08]	0.76*** [0.69–0.83]	1.39 [0.71–2.71]
Q4 (highest affluence)	0.9* [0.83–0.98]	0.77*** [0.7–0.85]	0.83*** [0.78–0.89]	1.04 [0.96–1.12]	0.87** [0.8–0.95]	0.7*** [0.65–0.76]	0.63 [0.23–1.72]
Neighborhood disadvantage							
Q1 (ref.)	Ref.	Ref.	Ref.	Ref.	Ref.	Ref.	Ref.
Q2	0.97 [0.93–1.02]	1.1+ [1–1.21]	1.12*** [1.05–1.19]	0.99 [0.9–1.07]	1.09*** [1.04–1.15]	1.03 [0.95–1.12]	1.86 [0.7–4.92]
Q3	1.02 [0.98–1.06]	1.16** [1.05–1.29]	1.21*** [1.1–1.32]	0.97 [0.88–1.06]	1.11** [1.04–1.18]	1.09*** [1.05–1.14]	2.53+ [0.89–7.14]
Q4 (highest disadvantage)	1.12*** [1.09–1.15]	1.23*** [1.13–1.35]	1.26*** [1.19–1.33]	0.96 [0.87–1.05]	1.16*** [1.08–1.24]	1.27*** [1.21–1.34]	2.86* [1.02–7.98]
Neighborhood population density	1 [1–1]	1 [1–1]	1+ [1–1]	1 [1–1]	1 [1–1]	1*** [1–1]	1 [1–1]
Political partisanship (10% increase in votes cast for Republican candidates in 2018 and the six years before)	1.03*** [1.03–1.04]	1.12*** [1.08–1.17]	0.98 [0.96–1]	1.01 [0.99–1.03]	1.01 [0.99–1.03]	0.81+ [0.64–1.02]	0.71 [0.33–1.55]
Rural-urban commuting area codes							
Metropolitan (ref.)	Ref.	Ref.	Ref.	Ref.	Ref.	Ref.	Ref.
Micropolitan	1.03 [0.98–1.08]	1.04 [0.92–1.19]	1.04 [0.83–1.29]	0.92** [0.86–0.98]	1.04 [0.95–1.14]	1 [1–1]	2.54 [0.65–9.89]
Small town	1.14*** [1.1–1.19]	1.05 [0.91–1.2]	1.01 [0.82–1.24]	0.85*** [0.[0.79–0.92]	0.89* [0.81–0.98]	1 [1–1]	2.29 [0.58–9.1]
Rural	1 [1–1]	1.01 [0.9–1.13]	0.98 [0.78–1.23]	0.76*** [0.7–0.81]	0.92* [0.85–0.99]	0.41*** [0.36–0.47]	1.74 [0.46–6.66]

+*p* < 0.10, *p < 0.05, **p < 0.01, ***p < 0.001.

**Table 3:** Results of the univariate regression results estimating the association between each neighborhood factor and neighborhood COVID-19 burden for the Southwestern United States

Southwest			
	Arizona	New Mexico	Oklahoma
	IRR [95% CI]	IRR [95% CI]	IRR [95% CI]
Neighborhood characteristic			
Neighborhood affluence			
Q1 (ref.)	Ref.	Ref.	Ref.
Q2	1.01 [0.92–1.1]	1 [0.87–1.13]	0.99 [0.94–1.05]
Q3	0.93 [0.83–1.06]	0.86* [0.75–0.99]	1.05 [0.98–1.12]
Q4 (highest affluence)	0.91 [0.78–1.05]	0.71*** [0.62–0.82]	1.09** [1.02–1.17]
Neighborhood disadvantage			
Q1 (ref.)	Ref.	Ref.	Ref.
Q2	1.09 [0.97–1.22]	1.12*** [1.05–1.2]	0.92*** [0.89–0.96]
Q3	1.15*** [1.07–1.23]	1.25*** [1.16–1.34]	0.89*** [0.85–0.94]
Q4 (highest disadvantage)	1.16* [1.03–1.31]	1.31*** [1.18–1.44]	0.88*** [0.82–0.95]
Neighborhood population density	1 [1–1]	1 [1–1]	1* [1–1]
Political partisanship (% of votes cast for Republican candidates in 2018 and the 6 years before)	1.04 [0.94–1.16]	1.06*** [1.03–1.09]	1 [0.97–1.04]
Rural-urban commuting area codes			
Metropolitan (ref.)	Ref.	Ref.	Ref.
Micropolitan	1.11 [0.95–1.28]		0.99 [0.93–1.06]
Small town	0.66** [0.51–0.86]		0.97 [0.91–1.04]
Rural	0.55*** [0.4–0.74]		0.92* [0.86–0.98]

+*p* < 0.10, *p < 0.05, **p < 0.01, ***p < 0.001.

**Table 4:** Results of the univariate regression results estimating the association between each neighborhood factor and neighborhood COVID-19 burden for the Western United States

West		
	Nevada	Oregon
	IRR [95% CI]	IRR [95% CI]
Neighborhood characteristic		
Neighborhood affluence		
Q1 (ref.)	Ref.	Ref.
Q2	1.02 [0.98–1.05]	0.9* [0.83–0.98]
Q3	0.97 [0.9–1.05]	0.83*** [0.77–0.89]
Q4 (highest affluence)	0.91*** [0.88–0.95]	0.73*** [0.65–0.81]
Neighborhood disadvantage		
Q1 (ref.)	Ref.	Ref.
Q2	1 [0.94–1.06]	1.12+ [1–1.25]
Q3	1.09*** [1.04–1.15]	1.25*** [1.17–1.34]
Q4 (highest disadvantage)	1.15*** [1.07–1.22]	1.4*** [1.29–1.52]
Neighborhood population density	1 [1–1]	1** [1–1]
Political partisanship (% of votes cast for Republican candidates in 2018 and the six years before)	0.87*** [0.82–0.91]	1.1*** [1.04–1.16]
Rural-urban commuting area code**s**		
Metropolitan (ref.)	Ref.	Ref.
Micropolitan	0.72*** [0.62–0.83]	1.19** [1.05–1.36]
Small town	0.51*** [0.42–0.62]	1.08 [0.89–1.31]
Rural	0.51** [0.32–0.83]	1.01 [0.84–1.21]

+*p* < 0.10, *p < 0.05, **p < 0.01, ***p < 0.001.

**Table 5:** Results of the univariate regression results estimating the association between each neighborhood factor and neighborhood COVID-19 burden for the Southeastern United States

Southeast				
	Florida	Louisiana	North Carolina	Virginia
	IRR [95% CI]	IRR [95% CI]	IRR [95% CI]	IRR [95% CI]
Neighborhood characteristic				
Neighborhood affluence				
Q1 (ref.)	Ref.	Ref.	Ref.	Ref.
Q2	0.94 [0.88–1.02]	0.99 [0.95–1.03]	0.99 [0.96–1.03]	1.01 [0.97–1.05]
Q3	0.89* [0.8–0.99]	1.03 [0.98–1.07]	0.98 [0.94–1.03]	0.96* [0.93–1]
Q4 (highest affluence)	0.9* [0.81–1]	1.05* [1–1.11]	0.92** [0.86–0.98]	0.85*** [0.78–0.93]
Neighborhood disadvantage				
Q1 (ref.)	Ref.	Ref.	Ref.	Ref.
Q2	1.08+ [0.99–1.18]	0.99 [0.96–1.03]	1.07*** [1.03–1.11]	1.1** [1.04–1.17]
Q3	1.18** [1.07–1.31]	0.93** [0.89–0.97]	1.09*** [1.04–1.15]	1.2*** [1.1–1.32]
Q4 (highest disadvantage)	1.33*** [1.14–1.56]	0.96* [0.93–1]	1.13*** [1.08–1.18]	1.2** [1.08–1.34]
Neighborhood population density	1*** [1–1]	1 [1–1]	1* [1–1]	1+ [1–1]
Political partisanship (% of votes cast for Republican candidates in 2018 and the six years before)	0.87* [0.78–0.97]	1.01 [0.99–1.02]	1.02+ [1–1.04]	1.04** [1.01–1.07]
Rural-urban commuting area codes				
Metropolitan (ref.)	Ref.	Ref.	Ref.	Ref.
Micropolitan	0.92 [0.74–1.14]	0.92* [0.86–0.99]	1.02 [0.96–1.07]	1.17*** [1.08–1.26]
Small town	1.03 [0.85–1.26]	0.95 [0.84–1.07]	0.96 [0.89–1.04]	1.12* [1.03–1.22]
Rural	1.04 [0.78–1.37]	0.81*** [0.72–0.91]	0.94 [0.87–1.01]	1.05 [0.96–1.15]

+*p* < 0.10, *p < 0.05, **p < 0.01, ***p < 0.001.

**Table 6:** Results of the univariate regression results estimating the association between each neighborhood factor and neighborhood COVID-19 burden for the Midwestern United States

Midwest					
	Illinois	Indiana	Minnesota	Ohio	Wisconsin
	IRR [95% CI]	IRR [95% CI]	IRR [95% CI]	IRR [95% CI]	IRR [95% CI]
Neighborhood characteristic					
Neighborhood affluence					
Q1 (ref.)	Ref.	Ref.	Ref.	Ref.	Ref.
Q2	1.08* [1.01–1.16]	1.12** [1.04–1.21]	1.05+ [1–1.1]	1.08*** [1.03–1.12]	0.95*** [0.93–0.98]
Q3	1.07* [1–1.14]	1.11** [1.03–1.19]	1.05+ [1–1.1]	1.09*** [1.05–1.13]	0.98 [0.95–1.01]
Q4 (highest affluence)	1.02 [0.96–1.09]	1.19*** [1.09–1.3]	1.02 [0.97–1.07]	1.07*** [1.04–1.1]	0.97 [0.93–1.02]
Neighborhood disadvantage					
Q1 (ref.)	Ref.	Ref.	Ref.	Ref.	Ref.
Q2	1.02 [1–1.04]	0.97 [0.92–1.03]	1.02 [0.98–1.07]	1.05*** [1.02–1.08]	0.98 [0.95–1.01]
Q3	1.03 [0.96–1.11]	0.96 [0.89–1.04]	1 [0.97–1.03]	1.05** [1.02–1.09]	1.02 [0.99–1.06]
Q4 (highest disadvantage)	0.99 [0.89–1.1]	0.9** [0.84–0.96]	1.05* [1.01–1.09]	1.04* [1–1.09]	1.06** [1.02–1.1]
Neighborhood population density	1*** [1–1]	1*** [1–1]	1*** [1–1]	1*** [1–1]	1*** [1–1]
Political partisanship (% of votes cast for Republican candidates in 2018 and the six years before)	1.07*** [1.05–1.08]	1.06*** [1.03–1.08]	1.03*** [1.01–1.05]	1.02** [1.01–1.03]	1 [0.99–1.02]
Rural-urban commuting area codes					
Metropolitan (ref.)	Ref.	Ref.	Ref.	Ref.	Ref.
Micropolitan	1.16*** [1.07–1.25]	1.06 [0.99–1.13]	1 [0.94–1.06]	1.05* [1.01–1.1]	0.97 [0.92–1.02]
Small town	1.21*** [1.13–1.3]	0.97 [0.9–1.05]	0.96 [0.92–1.02]	1.02 [0.98–1.06]	0.95** [0.91–0.99]
Rural	1.07+ [1–1.15]	0.9 [0.8–1.02]	0.9*** [0.86–0.95]	0.82** [0.7–0.95]	0.85*** [0.81–0.9]

+*p* < 0.10, *p < 0.05, **p < 0.01, ***p < 0.001.

In 62% of states, we observe a positive, statistically significant correlation between neighborhood disadvantage and COVID-19 burden wherein higher quartiles of neighborhood disadvantage (i.e., those more disadvantaged) are associated with a higher rate of COVID-19 (Tables 2–6 and S2). For example, in the highest quartile (Q4) of disadvantaged neighborhoods in Vermont, the rate is 2.86 (95% CI: 1.02–7.98) times that of neighborhoods in the lowest quartile of disadvantage (Q1, i.e., those least disadvantaged). In Oregon, neighborhoods in the highest quartile of disadvantage (Q4) have 1.40 (95% CI: 1.29–1.52) times the rate of COVID-19 compared to neighborhoods in the lowest quartile of disadvantage (Q1).

We do not see any consistent patterns among the correlations with population density (Tables 2–6 and S2).

We also examine correlations between political partisanship and COVID-19. For county-level political partisanship, we find that in 38% of states, there is a statistically significant, positive correlation between county-level political partisanship and neighborhood COVID-19 burden (Table S2). That is, neighborhoods in which a greater proportion of the votes were cast for Republican candidates in senate and presidential races from 2012-2018 have a higher burden of COVID-19 at the neighborhood-level. In univariate analyses, we operationalized the partisanship variable such that a one-unit increase in the political partisanship variable is equivalent to a 10% increase in Republican votes (Tables 2–6). For example, in Maine, a 10% increase in Republican votes cast is associated with a 12% increase in the COVID-19 rates in neighborhoods (*b* = 1.12, 95% CI: 1.08–1.17). In Illinois, a 10% increase in Republican votes cast is associated with a 7% increase in the COVID-19 rate (*b* = 1.07, 95% CI: 1.05-1.08).

However, for several states (24%) we simultaneously observe a strong, negative correlation between county-level political partisanship and COVID-19 burden (Tables 2–6). In Nevada, for example, a 10% increase in the percentage of votes cast for Republican candidates is associated with a 13% lower rate of COVID-19 (*b* = 0.87, 95% CI: 0.82–0.91). Similarly, in Florida, a 10% increase in the percentage of votes cast for Republican candidates is associated with a 13% lower rate of COVID-19 (*b* = 0.87, 95% CI: 0.78–0.97).

Finally, we observe a statistically significant, negative correlation between the RUCA categories and COVID-19 burden for 48% of states (Tables 2–6, Table S2). In Rhode Island, neighborhoods classified as rural have a 59% lower rate of COVID-19 compared to neighborhoods classified as metropolitan (*b* = 0.41 95% CI: 0.36–0.47). In Arizona, neighborhoods classified as rural have a 45% lower rate of COVID-19 compared to neighborhoods classified as metropolitan (*b* = 0.55, 95% CI: 0.4–0.74) (Table 2). While there are several states for whom we observe a positive correlation between the RUCA and COVID-19 burden, the correlations are small and not statistically significant.

## Discussion

Our results are the first findings from the COVID Neighborhood Project (CONEP), a data effort designed to collate spatially-referenced COVID-19 data at the neighborhood-level across the U.S. The findings from this study are suggestive that the state context may matter for determining the distribution of COVID-19 across neighborhoods. For some states, there is wide variation in the neighborhood COVID-19 burden (i.e., Maine, Vermont, Pennsylvania), a signal that some neighborhoods account for a disproportionate burden of COVID-19 cases compared to other neighborhoods in the same state. In contrast, for other states, the burden of COVID-19 is more homogenously distributed across neighborhoods (i.e., Delaware, Wisconsin, Louisiana). Though the results from the current investigation are preliminary and descriptive in nature, they underscore that local neighborhood patterns and dynamics play an underappreciated role in the distribution and intensity of COVID-19 burden and the long-term sequalae that will be endured. These findings also hint at the complex relationship between state- and local-based policies, neighborhood features, and the myriad ways in which the interaction of these area-based forces may have influenced the COVID-19 burden felt by millions. Indeed, policy makers should be wary in crafting a one-size-fits-all approach for pandemic mitigation and recovery efforts. Our results suggest that among the states included in this analysis, there may be no single, unifying story to describe how COVID-19 has been distributed across neighborhoods within states in the U.S.

Our findings are not only consistent with other investigations conducted in the U.S. but in various global settings as well. For example, in a study of the spatiotemporal dynamics of the spread of COVID-19 in Brazil in 2020, Castro et al, found a diverse array of factors explained how the virus was distributed across various states within Brazil. There were complex interactions between state- and local-level policy, socioeconomic, and political environments that shaped the distribution of COVID-19 both within- and between states^71^. In a study examining trends in COVID-related intensive care unit admissions in Sweden, Kawalerowicz et al. found that individuals living in neighborhoods that were classified as rural and disadvantaged had a higher risk of ICU admission^72^. Studies in the United Kingdom (U.K.) have also reported findings that neighborhood deprivation has indeed shaped the distribution of COVID-19 burden but cannot be divorced from individual-level attributes of racial and ethnic identification and socioeconomic conditions^73,74^. The studies above demonstrate that the complex social and environmental dynamics shaping the COVID-19 pandemic have been observed across multiple country contexts. We resonate with Bollyky et al. in that inequities on the basis of race/ethnicity and socioecomic status and political polarization including politicizing of the pandemic are not unique to the U.S. context^1^. Collectively, therefore, findings from mutiple countries, including our own, may begin to shed light on how the increasingly complicated socioeconomic, political, and environmental context shapes infectious disease spread.

Historically, there have been numerous studies relating aspects of the social and built neighborhood environment to infectious disease in the U.S.^56,75–79^. For many of these studies, a traditional paradigm prevails in which more disadvantaged neighborhoods experience a higher burden of disease^55,56,76^. While our study did not apply a causal framework, our initial findings support the paradigm of an association between disadvantage and higher infectious burden but only for some states. Historically accepted patterns of the relationship between the neighborhood environment and infectious disease burden are challenged in other states; this paradigm is more nuanced and complex than previously imagined. While we are limited by the data we have been able to collect thus far both in geographic scope (i.e., not all 50 states) and other key confounding variables (e.g., neighborhood vaccination rates, access to vaccination sites, access to health facilities, and test positivity rates at the local area level), these broad signals offer a window into the complicated relationship that exists between aspects of the social and built neighborhood environment, state and local policy, and COVID-19 burden. As shown in other studies, there are state-specific behaviors and policies that have shaped the variation in the COVID-19 burden across the U.S.^64,80–83^. Our findings lay the groundwork for future work that can build off of our results to more explicitly examine and test how state-specific behaviors interact with local area-level concentrated disadvantage or affluence.

One such example of this interaction is with regards to rural populations. In one of the few studies that focused exclusively on understanding the impacts of COVID-19 on rural populations in the U.S., Mueller et al. found extensive reports of the negative consequences from COVID-19 among rural populations including in unemployment rates, perceptions of the local economy, and impact on their overall lives and mental wellbeing^52^. They reported a 9.7 percentage point increase in the 2020 unemployment rate in the rural communities included in their sample compared to the year before the pandemic^52^. This contrasts with a national increase in the unemployment rate of only 7.4 percentage points. With increasing urbanization, rural populations represent a unique, and diverse community in the U.S., one particularly vulnerable to long-term social and economic consequences from COVID-19 and whom have often been overlooked in the pandemic response^84–86^.

There were multiple strengths to the current study. To the best of our knowledge, we are among one of the first data collation efforts to attempt to collect in-depth, spatially-referenced case data at the neighborhood-level for the entire U.S., and the only to link these spatially-referenced case data at the census tract or ZIP code level with features of the neighborhood built and social environment from the NANDA repository. Our results have implications for both public health practice and policy and for how we understand the health of the U.S. population going forward. They also lay the groundwork for developing effective prevention and mitigation strategies to address continuing inequities in pandemic-related consequences. These data provide the foundation for future studies focused on the impacts of pandemic-related changes to the neighborhood environment and population health trends, and highlight the need to gather robust neighborhood-level disease, long-term sequelae, and environmental metrics for COVID-19 across the U.S. given the heterogeneity we observed by state.

While this study currently advances our understanding of how COVID-19 has differentially impacted neighborhoods across 21 states in the U.S., there are limitations that should be considered when interpreting these results and that may also guide future investigations. Given the emergency nature of the pandemic and the independence of state public health institutions from federal jurisdiction, each state set up surveillance systems differently, including defining different inclusion criteria and methods for counting cases, and in the selection of the types of health facilities surveilled (22-23). For example, if a state only included laboratory-confirmed cases, their case counts were likely an underestimate of the true case counts in a population, however, this was not standardized. Laboratory-confirmed case counts may miss cases identified through at-home testing which increased in the later stages of the pandemic. Further, state case data often differed in geographic and temporal coverage, also in part due to the rise in at home testing beginning from late 2021 onwards and other advances or policy changes, which made more nuanced comparisons by state difficult. While examining trends in the aggregate may mitigate some of the state-to-state differences, comparisons across states should still be made with caution; accounting for the decrease in case count accuracy over time is difficult even for the most robust surveillance teams.

Moreover, we only have data on incident infections. Thus, the case data could reflect repeat infections occurring in the same person. However, we nonetheless can detect neighborhoods that have had the highest COVID-19 burden. Additionally, we do not have information on co-morbid conditions that occurred with COVID-19 infections, which may serve as important underlying drivers of COVID-19 infections and occur more frequently among structurally disadvantaged populations^87^. Future analyses should incorporate data on the prevalence of key co-morbid conditions into statistical models.

Importantly, as state case counts reflect not only the availability of testing facilities and infrastructure in place to report positive test results but access to such facilities as well, the current results may best be viewed as a proxy indicator of the true underlying trends in COVID-19 case data. In future studies, we plan to collate data on testing sites, test positivity rates, other indicators of the burden of COVID-19 (e.g., wastewater surveillance), and mitigation strategies (e.g., vaccination history) over time to create a more accurate indicator of COVID-19 burden.

Despite the limitations, these findings outline the necessary steps towards a more comprehensive data collation effort and possibly the foundation for a concerted surveillance system across the U.S. Compared to other U.S. spatially-referenced COVID-19 datasets like the Centers for Disease Control and Prevention COVID-19 Data Tracker or the Johns Hopkins University Coronavirus Resource Center, which are both limited to county-level data, harnessing a repository like CONEP will allow researchers and the public to examine more local level trends, while simultaneously accounting for the heterogeneity that exists within counties and across the urban to rural expanse.

As this is the first investigation in a series of studies stemming from CONEP, we will continue to build the CONEP repository to cover all 50 states and build in more contextual data at the state, county, and local levels. Also, it is critical to note that obtaining local-level, finer-scale spatial data is difficult and should involve a deeper discussion of ethics. Calling on public health practitioners, who are often burdened with other real-time tasks, to collect such data may not always allow for the priority it should be given until systems are automated. Still, gathering these data is essential for monitoring health and social well-being in the U.S.

## Conclusion

The inequitable distribution of COVID-19 across neighborhoods in the U.S. has consequences both for the current health and economic wellbeing of the American population as well as for the future of population health in the U.S., and specifically population health inequities^88,89^. Therefore, it is imperative that we interrogate patterns and trends at the local neighborhood-level. Our results represent an initial step to document the complex ways in which the neighborhood social environment may be related to COVID-19 burden and highlight the importance of the local-level for determining patterns of disease risk and resilience. While the traditional paradigm in infectious disease research has long held that poverty increases infectious disease burden, our findings highlight state-level variation, and that a one-size-fits-all approach will not address the unique patterns observed across states in the U.S. By leveraging fine-scale spatially-referenced case data, our findings enhance our understanding of the neighborhood social environment and COVID-19 burden and underscore the continued need for nationwide data for neighborhoods across all states in order to adequately improve the health of populations.

### Supplementary information

Supplementary Information

## Data Availability

All of the individual state-level data are publicly available. We have included information regarding how to obtain the data for each state in the supplement including the necessary links (see Table S3). Efforts are currently underway to work with each individual health department, adhering to their specific data protection procedures, to compile all of the state-level data into a single database that will be publicly available through ICPSR. Further, in the current investigation, we analyzed each state separately and thus downloading each state individually will allow for replicability. For any questions or updates on the process of building a single, national database, please contact the corresponding author. The NANDA data are available through ICPSR and instructions for accessing the NANDA data and the data itself are available at https://www.icpsr.umich.edu/web/ICPSR/series/1920.

## Code availability

The code used to generate the results are available at 10.5281/zenodo.10606856^90^.
