# Supplementary material for: State variation in neighborhood COVID-19 burden across the United States
Source: Commun Med (Lond). 2024 Mar 1;4:36. doi: 10.1038/s43856-024-00459-1 (PMC10907669; doi:10.1038/s43856-024-00459-1)
Supplement: Supplementary file 1 — Supplementary Information [file 43856_2024_459_MOESM1_ESM.pdf]

Supplementary Information

Title: State Variation in Neighborhood COVID-19 Burden Across the United States

Authors:

Grace A Noppert[1*], Philippa Clarke[1], Andrew Hoover[1], John Kubale[1], Robert Melendez[1], Kate Duchowny[1] & Sonia T Hegde[2]

[1] Institute for Social Research, University of Michigan, USA
[2] Department of Epidemiology, Johns Hopkins University, USA
[*] Indicates corresponding author

Correspondence:
Grace A Noppert
Institute for Social Research
University of Michigan
426 Thompson St.
Ann Arbor, MI 48103 USA
Email: gnoppert@umich.edu
Phone: +001 734-764-8354

Figures

Supplementary Figure 1. Map showing the states currently included in the COVID Neighborhood Project (CONEP). The case counts per 100,000 population categorized into deciles for all 21 states are depicted. The case counts refer to cumulative case counts for the time period. Color gradations refer to higher deciles of case counts.

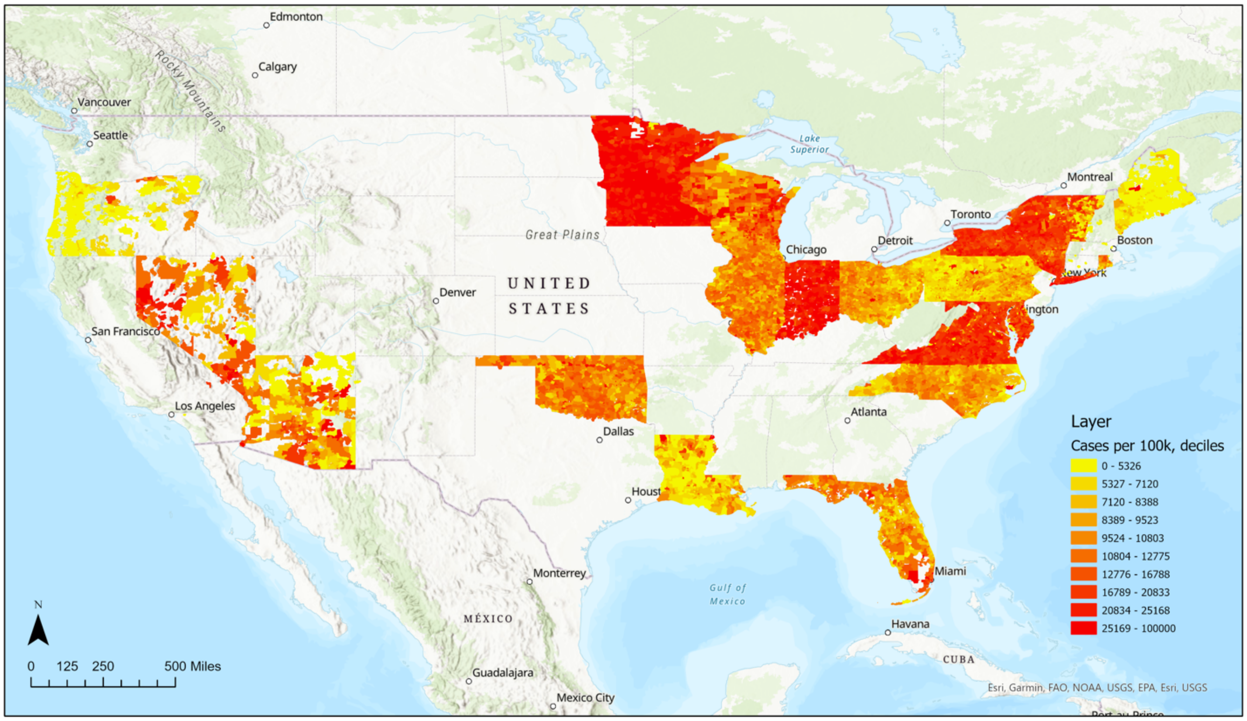

Supplementary Figure 2. Map showing the case counts per 100,000 persons for Oregon, Nevada, and Arizona. The case counts per 100,000 population categorized into deciles for all 21 states are depicted. The case counts refer to cumulative case counts for the time period. Color gradations refer to higher deciles of case counts.

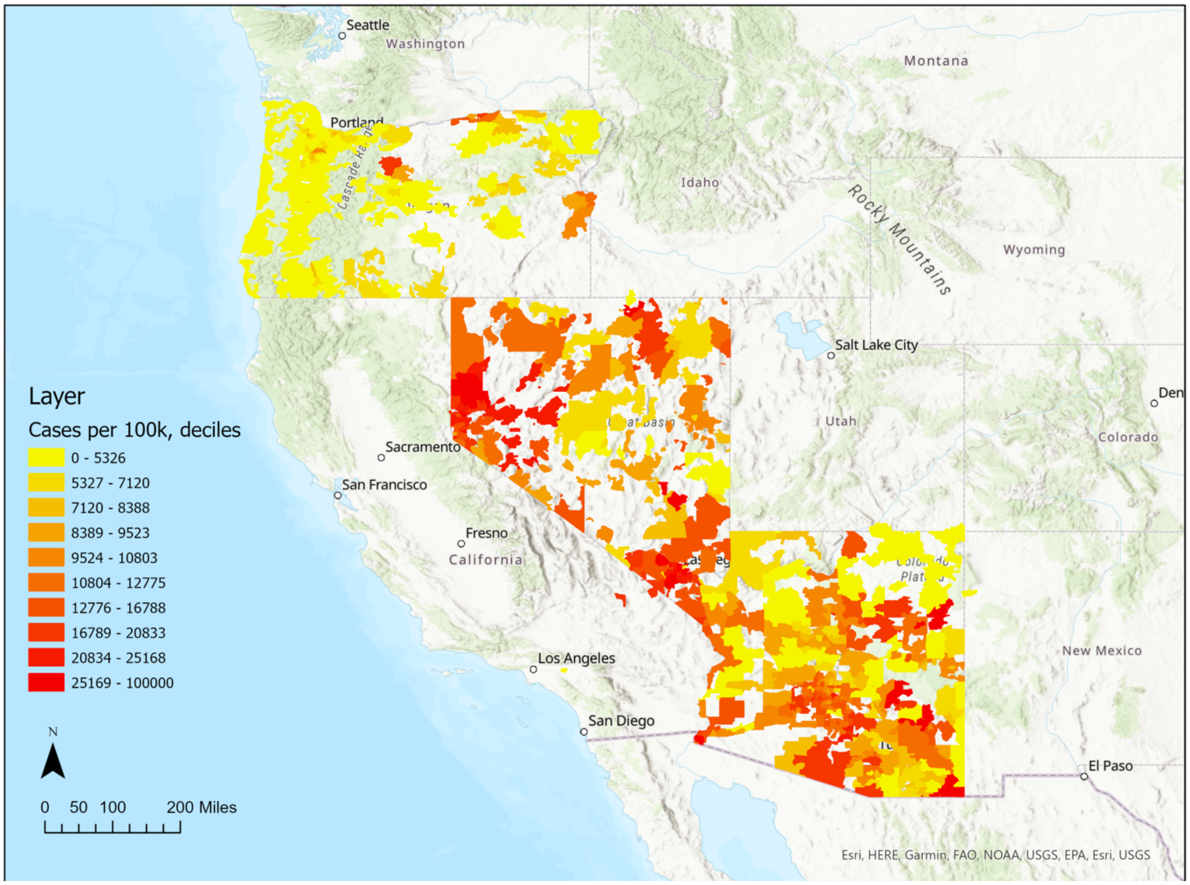

Supplementary Figure 3. Map showing the case counts per 100,000 persons for Maine, New York, Rhode Island, and Pennsylvania. The case counts per 100,000 population categorized into deciles for all 21 states are depicted. The case counts refer to cumulative case counts for the time period. Color gradations refer to higher deciles of case counts.

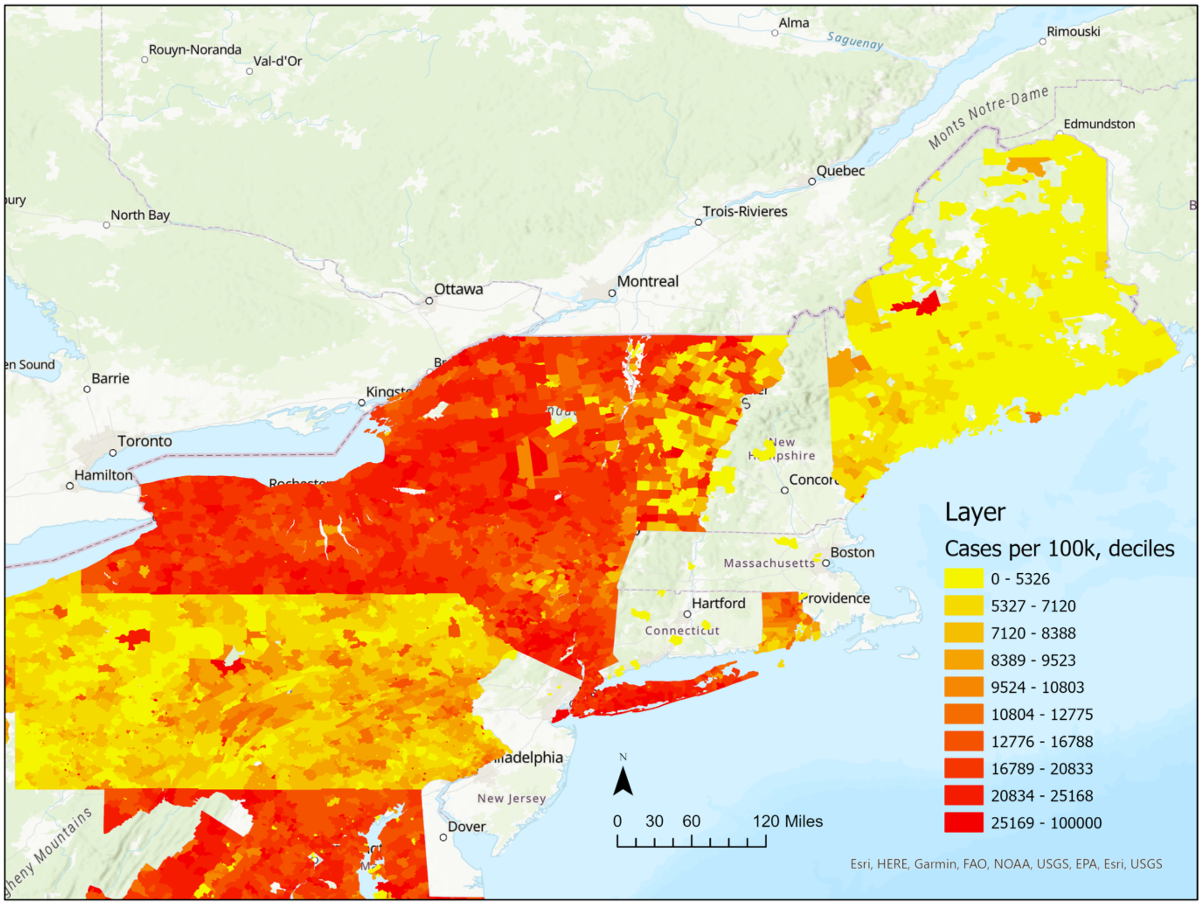

Tables

Supplementary Table 1. Number of spatial units in each state missing data.

State	Spatial Resolution	Number of Spatial Units Missing Data/ Total Spatial Units in the State**	Date Collected Through
	**Northeast**		
Delaware	Census tract	38/214	6/2/2022
Maine	ZCTA	38/427	5/8/2022
Maryland	ZCTA	21/464	7/11/2022
New York	ZCTA	0/1,753	9/18/2022
Pennsylvania	ZCTA	50/1,783	5/20/2022
Rhode Island	Census tract	0/77	7/31/2022
Vermont	ZCTA	0/254	6/4/2022
	**Southwest**		
Arizona	ZCTA	48/397	6/1/2022
New Mexico	Census tract	4/603	6/1/2022
Oklahoma	ZCTA	20/646	5/25/2022
	**West**		
Nevada	ZCTA	1/171	7/3/2022
Oregon	ZCTA	143/415	6/3/2022
	**Southeast**		
Florida	ZCTA	36/976	5/23/2021
Louisiana	Census tract	10/1,127	5/18/2022
North Carolina	ZCTA	41/801	5/26/2022
Virginia	ZCTA	0/891	5/23/2022
	**Midwest**		
Illinois	ZCTA	31/1,381	7/12/2022
Indiana	ZCTA	251/771	5/19/2022
Minnesota	ZCTA	8/880	7/31/2022
Ohio	ZCTA	52/1,188	5/20/2022
Wisconsin	Census tract	0/1,392	9/25/2022

**Indicates the total number of spatial units with population that are missing values

Supplementary Table 2. Correlation coefficients estimating the correlation between each neighborhood characteristics and COVID-19 cases per 10,000 population. Correlations were estimated for each state separately.

	**Arizona**	**Delaware**	**Florida**	**Illinois**	**Indiana**	**Louisiana**
	**ρ** Cases per 10k	**ρ** Cases per 10k	**ρ** Cases per 10k	**ρ** Cases per 10k	**ρ** Cases per 10k	**ρ** Cases per 10k
**Neighborhood Characteristics**						
Neighborhood Affluence	.092+	-.2171**	0.0319	-0.0422	.2972***	0.0286
Neighborhood Disadvantage	0.0235	.2218**	.2787***	-.0963***	-.241***	-0.0321
Neighborhood Population Density	.2373***	-0.0151	.3629***	-.1278***	-.2184***	-.0725*
County-Level Political Partisanship	0.0863	.186*	-.2786***	.2631***	.1802***	.0615*
RUCA codes: Metropolitan(low)-Rural(high)	-.3718***	.1423+	-0.0191	.0961***	-.0963*	-.164***

+ p<0.10, * p<0.05, ** p<0.01, *** p<0.001

	**Maine**	**Maryland**	**Minnesota**	**New Mexico**	**North Carolina**	**Nevada**
	**ρ** Cases per 10k	**ρ** Cases per 10k	**ρ** Cases per 10k	**ρ** Cases per 10k	**ρ** Cases per 10k	**ρ** Cases per 10k
**Neighborhood Characteristics**						
Neighborhood Affluence	-.2187***	-.1946***	.0589+	-.3187***	-.1505***	-0.0221
Neighborhood Disadvantage	.1942***	.1569***	.1566***	.1486***	.1907***	.3112***
Neighborhood Population Density	0.023	.0898+	.0607+	-0.0232	0.0598	.3476***
County-Level Political Partisanship	.1354**	-0.0268	0.0079	.1852***	0.0587	-.536***
RUCA codes: Metropolitan(low)-Rural(high)	-0.0519	-0.042	-.1928***		-.1624***	-.4734***

+ p<0.10, * p<0.05, ** p<0.01, *** p<0.001

	New York	Ohio	Oklahoma	Oregon	Pennsylvania	Rhode Island	Vermont	Virginia	Wisconsin
	**ρ** Cases per 10k	**ρ** Cases per 10k	**ρ** Cases per 10k	**ρ** Cases per 10k	**ρ** Cases per 10k	**ρ** Cases per 10k	**ρ** Cases per 10k	**ρ** Cases per 10k	**ρ** Cases per 10k
**Neighborhood Characteristics**									
Neighborhood Affluence	0.0298	.0661*	0.064	-.2475***	-.0707**	-.6271***	-.197***	-0.0317	-.0828**
Neighborhood Disadvantage	.0746**	0.0042	-0.0269	.3275***	-0.0072	.4538***	.1438*	0.0212	.0909***
Neighborhood Population Density	.0402+	-.0776**	-0.0036	-0.0358	-0.0028	.3928***	.1367*	-.1027**	.1785***
County-Level Political Partisanship	-.064**	.0503+	-.1332***	.271***	-0.0098	-.3588***	-0.0105	.1503***	0.0035
RUCA codes: Metropolitan(low)-Rural(high)	-.1899***	-0.029	-.1289**	0.0333	-0.0311	-.1603*	-0.0434	.0807*	-.2561***

+ p<0.10, * p<0.05, ** p<0.01, *** p<0.001

Supplementary Table 3. Information on how data from each state was collected and the necessary steps to go through to access the data.

State	Health Department	Spacial Granularity Of Data Collected	Time Period Granularity Of Data Collected	Date Data Was Retreived	How Data Was Retreived	Data Was Retreived Through FOIA(Like) Request	URL
Arizona	Arizona Department of Health Services	Zip code	One-time	6/1/2022	Public download	No	https://www.azdhs.gov/covid19/data/index.php#zipcode
Delaware	Delaware Department of Health and Social Services	Census tract	Daily	6/3/2022	Public download	No	https://myhealthycommunity.dhss.delaware.gov/locations/state#<br>1. Select COVID data of interest<br>2. Follow resulting directions to download data
Florida	Florida Department of Health	Zip code	One-time	5/23/2021	Public download	No	Reach out to investigator for data<br>Note. Data is not publicly available for download past 6/03/21
Illinois	Illinois Department of Public Health	Zip code	One-time	7/12/2022	download	No	https://dph.illinois.gov/covid19/data.html
Indiana	Indiana Department of Health	Zip code	One-time	3/21/2022	Public download	No	https://hub.mph.in.gov/dataset/covid-19-cases-by-zip<br>Note. Data has a quality issue and is unable to download past 3/24/22
Louisiana	Louisiana Department of Health	Census tract	Weekly	5/26/2022	Public download	No	https://ldh.la.gov/Coronavirus/
Maine	Maine Department of Health and Human Services	Zip code	One-time	5/19/2022	Public download	No	https://www.maine.gov/dhhs/mecdc/infectious-disease/epi/airborne/coronavirus/data.shtml
Maryland	Maryland Department of Health	Zip code	Daily	7/12/2022	Public download	No	https://coronavirus.maryland.gov/
Minnesota	Minnesota Department Of Health	Zip code	Monthly	8/2/2022	Email communication	Yes/No	https://www.health.state.mn.us/data/datapractices/index.html<br>1. Consider data request or data practices request<br>2. Follow corresponding information for request type picked
Nevada	Nevada Department of Health and Human Services	Zip code	Weekly	7/3/2022	Email communication	Yes	https://dhhs.nv.gov/About/PublicRecordsRequest/<br>1. Submit request through portal
New Mexico	New Mexico Department of Health	Census tract	Monthly	6/21/2022	Email communication	Yes	https://www.nmhealth.org/about/asd/cmo/ipra/<br>1. Submit request through portal
New York	New York State Department of Health	Zip Code	Daily	9/23/2022	Public download	No	https://health.data.ny.gov/Health/New-York-State-Statewide-COVID-19-Testing-by-Zip-C/e7te-hhb2/data
North Carolina	North Carolina Department of Health and Human Services	Zip code	One-time	5/26/2022	Public download	No	https://covid19.ncdhhs.gov/dashboard/cases-and-deaths
Ohio	Ohio Department of Health	Zip code	One-time	5/20/2022	Public download	No	https://coronavirus.ohio.gov/dashboards/key-metrics/cases-by-zipcode
Oklahoma	Oklahoma State Department of Health	Zip code	One-time	5/26/2022	Public download	No	https://oklahoma.gov/covid19/community-data.html
Oregon	Oregon Department of Human Services	Zip code	One-time	6/3/2022	Public download	No	https://public.tableau.com/app/profile/oregon.health.authority.covid.19/viz/OregonCOVID-19CasesbyZIPCode/OregonsCOVID-19CasesbyZipCode
Pennsylvania	Pennsylvania Department of Health	Zip code	One-time	5/20/2022	Public download	No	https://www.health.pa.gov/topics/disease/coronavirus/Pages/Cases.aspx
Rhode Island	Rhode Island Department of Health	Census tract	Monthly	7/31/2022	Email communication	No	https://health.ri.gov/data/diseases/<br>1. Click "Email Program" under "Get Data"
Vermont	Vermont Department of Health	Zip code	Weekly	6/10/2022	Email communication	Yes	https://www.healthvermont.gov/about-us/contact-us/public-records<br>1. Click "REQUEST FOR DATA"<br>2. Email request to resulting contact
Virginia	Virginia Department of Health	Zip code	One-time	5/26/2022	Public download	No	https://data.virginia.gov/Government/VDH-COVID-19-PublicUseDataset-ZIPCode/8bkr-zfqv
Wisconsin	Wisconsin Department of Health Services	Census tract	Daily	9/26/2022	Public download	No	https://data.dhsgis.wi.gov/

Notes: Links to download data or begin the process of retreiving it can be found under the URL column- any extra instructions necessary are outlined. Data are cumulatively collected as of the date of the first confirmed case in each state. The date the data was retreived does not always equal the date the data is reflective through.
